# Knowledge and Self-Reported Clinical Experience of Oral Potentially Malignant Disorders Among Dental Trainees and General Dentists in Saudi Arabia: A Cross-Sectional Study

**DOI:** 10.3390/healthcare14203463

**Published:** 2026-10-15

**Authors:** Asma Saleh Almeslet, Sultan Binalrimal, Malak Mohammed Al Osaimi, Cristalle Soman, Khalid Fadhi Almutairi, Fai Khalid Almusaytir, Taym Hadi Khormi, Ghadah Hamoud Jammali, Reem Fahad Alhamid, Sami Abdulaziz Alfahad, Fahdah Ahmed Aldahash, Baraa Issam Abdulrahman

**Affiliations:** 1 Department of Oral Maxillofacial Surgery and Diagnostic Sciences, College of Medicine and Dentistry, Riyadh Elm University, Riyadh 11681, Saudi Arabia; alosaimimy@gmail.com (M.M.A.O.); fahda.aldahash@riyadh.edu.sa (F.A.A.); baraa.abdulrahman@riyadh.edu.sa (B.I.A.); 2 Restorative Dentistry Department, College of Medicine and Dentistry, Riyadh Elm University, Riyadh 11681, Saudi Arabia; sultan@riyadh.edu.sa; 3 College of Dentistry, Majmaah University, Al Majma’ah 11952, Saudi Arabia; dr.artkhalid@gmail.com (K.F.A.); samialfahad219@gmail.com (S.A.A.); 4 College of Dentistry, King Saud University, Riyadh 11545, Saudi Arabia; fai.k.almusaytir@gmail.com (F.K.A.); jammali.ghada@gmail.com (G.H.J.); reemalhamid2@gmail.com (R.F.A.); 5 College of Dentistry, Jazan University, Jazan 45142, Saudi Arabia; taym.khormi@gmail.com

**Keywords:** oral potentially malignant disorders, oral cancer, early detection, oral medicine, diagnosis, biopsy, referral, dental education, continuing professional development, Saudi Arabia

## Abstract

**Highlights:**

**What are the main findings?**

• Using a reduced seven-item knowledge score that excluded five ambiguous items, 921 respondents with complete retained-item data correctly answered 3.93 ± 1.82 of seven items on average; gaps remained in recognition of oral submucous fibrosis and, on an item with acknowledged wording ambiguity, in recognition of legion risk.
• Self-reported clinical exposure was limited within this sample; 59.4% reported no previous detection of a suspicious lesion, 50.1% reported no previous referral, and 78.9% reported never having performed an oral mucosal biopsy; “do not remember” responses were analysed separately.

**What are the implications of the main findings?**

• The observed pattern identifies topics that may warrant further educational needs assessment; this study did not evaluate educational interventions.
• Between-group differences, particularly those involving institutional affiliation and qualification, should be interpreted cautiously because study site, cumulative clinical opportunity, and item interpretation could not be fully separated.

**Abstract:**

Background/Objectives: Oral potentially malignant disorders (OPMDs) may precede oral squamous cell carcinoma, making their timely recognition and referral important. This study assessed OPMD knowledge and self-reported clinical experience among dental trainees and general dental practitioners (GDPs) in Saudi Arabia. Methods: A multicentre cross-sectional online survey was conducted between July 2022 and June 2023 using convenience sampling through institutional networks in Riyadh, Al Majma’ah, and Jazan. Knowledge was summarised as a seven-item score (range 0 to 7) after five ambiguous items were excluded. Associated characteristics were examined using multivariable linear regression with robust standard errors. Results: Of 923 respondents, 921 had complete knowledge data, with a mean score of 3.93 ± 1.82. Leukoplakia was identified as the most common OPMD by 70.7%, whereas only 17.0% recognised the early symptoms of oral submucous fibrosis. Overall, 59.4% reported never having detected a suspicious lesion, 50.1% never having referred a patient, and 78.9% never having performed an oral mucosal biopsy. Male sex and GDP status were associated with higher scores, whereas older age and private-institution affiliation were associated with lower scores (all *p* < 0.001). After excluding 250 respondents whose training stage was not stated, only the associations with GDP status and age 26 to 30 years remained statistically significant. Conclusions: In this cross-sectional study, gaps in OPMD knowledge coexisted with limited self-reported clinical experience. These findings indicate associations only, do not establish causality, and may not be generalisable to all dentists in Saudi Arabia.

## 1. Introduction

Cancers of the lip and oral cavity remain a substantial global health burden. The GLOBOCAN 2022 estimates placed lip and oral cavity cancer among the leading malignancies in men in countries with a lower Human Development Index, with an age-standardised incidence rate of approximately 10 per 100,000 [1], and more than 90% of these tumours are oral squamous cell carcinomas [2,3]. Although the oral cavity is one of the few anatomical sites amenable to direct visual and tactile examination without specialised equipment, most cases are still diagnosed at an advanced stage [4]. Because stage at diagnosis is the strongest determinant of outcome, the interval between the appearance of a mucosal abnormality and its definitive diagnosis represents one of the most tractable points for clinical intervention [5].

A proportion of oral squamous cell carcinoma is preceded by clinically detectable mucosal alterations grouped under the term oral potentially malignant disorders (OPMDs). The 2020 consensus of the World Health Organization Collaborating Centre for Oral Cancer defines OPMDs as oral mucosal abnormalities associated with a statistically increased risk of developing oral cancer, and recognises entities including oral leukoplakia, erythroplakia, proliferative verrucous leukoplakia, oral submucous fibrosis, oral lichen planus and lichenoid lesions, and actinic cheilitis, among others [6,7]. Critically for clinical practice, the risk carried by these entities is not uniform; a meta-analysis of malignant transformation by subtype reported pooled estimates ranging from 1.4% for oral lichen planus and 9.5% for leukoplakia to 33.1% for erythroplakia and 49.5% for proliferative verrucous leukoplakia [8]. A clinician who recognises leukoplakia but does not appreciate that a smaller, less conspicuous red patch may carry substantially greater risk may therefore have difficulty stratifying lesion risk appropriately, however broad their general awareness of oral cancer.

Tobacco in its smoked and smokeless forms, areca nut and betel quid, and alcohol remain the dominant modifiable risk factors, acting synergistically when combined [9,10,11,12]. In the Kingdom of Saudi Arabia, the risk profile has regional particularities, including the use of shammah, a smokeless tobacco preparation prevalent in the southern regions, and comparatively low population-level alcohol consumption. A recent review of the Saudi literature concluded that data on the prevalence of OPMDs in the Kingdom remain sparse and methodologically heterogeneous, and the authors called for a standardised national approach to their detection and management [13].

Dental practitioners occupy the gatekeeping position in this pathway. Conventional oral examination performed by a trained clinician has acceptable diagnostic accuracy for clinically evident lesions, although reported sensitivity and specificity vary considerably with examiner training [14]. Effective gatekeeping, however, depends on a sequence of clinical actions rather than knowledge alone: performing a systematic mucosal examination, recognising a lesion as suspicious, applying an appropriate threshold for definitive assessment of an unexplained persistent lesion, and then either performing a biopsy or referring the patient. Failure at any single step limits the value of competence at the others.

A substantial body of survey research has examined oral cancer awareness among dentists and dental students in the Gulf region and internationally [15,16,17,18,19]. These studies consistently report incomplete knowledge, particularly regarding high-risk sites and lesions with the greatest malignant potential. A multicentre European study of final-year dental students assessed OPMD knowledge alongside clinical experience and attitudes to future education, reporting variation between countries together with considerable interest in further training [20]. Previous Saudi and regional studies have predominantly characterised knowledge and awareness [15,16,17,18,19]. Comparable multicentre evidence that combines dental trainees with practising general dentists and examines whether knowledge is accompanied by clinically relevant experience in lesion recognition, referral and biopsy remains limited in Saudi Arabia. Because continuing professional development resources are finite, identifying specific areas of uncertainty within sampled populations is of practical value to educators and regulators.

The present study was designed to assess knowledge and self-reported clinical experience regarding OPMDs among dental trainees and general dental practitioners recruited through selected Saudi institutional networks, to describe how these outcomes varied across participant characteristics, and to examine characteristics associated with a knowledge score. The objectives were descriptive and exploratory. We anticipated that knowledge and self-reported clinical activity would not be fully concordant, because previous lesion detection, referral, and biopsy are lifetime experiences that depend on clinical opportunity, training context and scope of practice as well as on knowledge. This expectation was not formulated as a prespecified statistical hypothesis and no test of the individual-level relationship between knowledge and reported experience was prespecified or performed. The wider objective was to identify topics that may warrant further educational needs assessment in oral medicine.

## 2. Materials and Methods

### 2.1. Study Design and Reporting

This was a multicentre, observational, questionnaire-based cross-sectional study conducted between July 2022 and June 2023. Participants were recruited from dental colleges and affiliated clinical facilities in Riyadh, Al Majma’ah, and Jazan, Saudi Arabia. The results are reported in accordance with the Strengthening the Reporting of Observational Studies in Epidemiology (STROBE) statement for cross-sectional studies [21]. The reporting of the web-based survey elements is guided, where applicable, by the Checklist for Reporting Results of Internet E-Surveys (CHERRIES) [22] and the Consensus-Based Checklist for Reporting of Survey Studies (CROSS) [23]. The completed STROBE checklist is provided as File S1, the completed CHERRIES checklist as File S2, and the completed CROSS checklist as File S3.

### 2.2. Participants and Sampling

Eligible participants were undergraduate dental students in the clinical years of study, dental interns, and general dental practitioners currently working in governmental or private facilities in Saudi Arabia. Dental specialists, including consultants and residents in oral medicine, oral and maxillofacial surgery, and oral pathology, were excluded, as were preclinical students who had not yet undertaken supervised patient care.

Non-probability convenience sampling was used. The questionnaire was constructed in Google Forms (Google LLC, Mountain View, CA, USA) and circulated through institutional electronic mailing lists and messaging groups serving eligible students, interns and practitioners at the participating institutions. Participation was voluntary and unremunerated. The form was configured to require a response to each survey item and responses could not be revised after submission. Nevertheless, the final analysis dataset contained isolated blank cells in B7, B8, B9, B11, B12, C1, C2, C4, and C5. The origin of these isolated blanks could not be reconstructed retrospectively from the available records. They were therefore treated as missing values and were neither imputed nor treated as response categories. Respondent sign-in was required, e-mail addresses were not retained in the analytical file, and the single-response restriction available in Google Forms was not enabled. The dataset was screened for duplicate submissions during data cleaning by comparing the available sign-in/submission information and response patterns, and duplicates identified at that stage were removed. The screening log was not carried into the final analytical file, so the number of submissions removed cannot be reconstructed. The analytical dataset comprised 923 records, of which 920 were complete across all analysed fields. The questionnaire did not record the respondent’s institution name, only government or private affiliation, so the number of respondents contributed by each centre could not be determined and institution-level clustering could not be modelled.

The survey link was disseminated through participating institutional networks throughout the recruitment period, but the membership, delivery, and viewing denominators of those networks were not available. A formal response rate and the magnitude of non-response bias therefore could not be estimated. No a priori sample-size calculation was performed; the adequacy of the achieved sample was therefore assessed retrospectively (Section 2.3). The resulting sample should be interpreted as a convenience sample from the participating networks rather than as representative of the national dental workforce.

### 2.3. Sample Size Estimation

Because no a priori sample-size calculation was performed before data collection, the adequacy of the achieved sample was evaluated retrospectively for the reported descriptive and multivariable analyses. For item-level proportions, a conservative calculation using the single-proportion formula n = Z^2^*p*(1 − *p*)/d^2^, assuming a proportion of 0.50, 95% confidence (Z = 1.96) and a 5% margin of error (d = 0.05), yielded a minimum sample of 385 respondents. For the multivariable linear regression with five predictor terms (sex, two age indicators, qualification, and institutional affiliation), 647 respondents were required to detect a small overall effect (Cohen’s f^2^ = 0.02, the conventional benchmark for a small effect) with 80% power at α = 0.05, calculated from the noncentral F distribution. The 921 complete cases available for the primary knowledge-score analysis exceeded these benchmarks. These retrospective calculations did not guide recruitment and serve only to place the achieved sample in context; they do not alter the non-probability nature of the sample or its limited generalisability, and estimates for smaller subgroups remain less precise (Section 4.6).

### 2.4. Questionnaire Development and Content

A questionnaire comprising 4 sociodemographic items, 12 knowledge items, and 5 clinical experience and perception items was assembled by adapting items from previously published questionnaires addressing oral cancer and OPMD awareness among dental populations [16,17,24,25]. The instrument was administered in English, which is the language of dental instruction and clinical record keeping at the participating institutions, and no translated version was used. Because the items were combined and adapted for the present population, content validity assessment and pilot testing were undertaken before administration.

The first part recorded four sociodemographic and professional characteristics: sex; age group (20–25, 26–30, or >30 years); recorded level (last-year dental student, dental intern, dental general practitioner, or ‘others’); and institutional affiliation (government or private). For the primary qualification analyses, all non-practitioner responses were grouped as trainees. Eligibility was restricted to clinical-year students, interns and general dental practitioners, and the survey was circulated through networks serving these groups (Section 2.2). Thus, respondents selecting ‘others’ are treated as trainees who did not identify their stage; this classification rests on the eligibility criteria and the distribution channels rather than on a recorded training stage and could not be verified from the data. Because the ‘others’ response does not identify a stage, 250 of the 722 trainees could not be allocated to a student or an intern group, so the student-versus-intern comparison was restricted to the 472 respondents who identified one of these two stages.

The second part contained 12 closed-ended knowledge items with a single designated correct response, covering the natural history of OPMDs and of epithelial dysplasia, the interval after which an unexplained oral lesion warrants definitive diagnostic assessment, the most frequent OPMD, the use of the term leukoplakia, the commonest site of oral erythroplakia, the lesion most likely to exhibit dysplasia or carcinoma, the subtype of lichen planus carrying the greatest malignant potential, the clinical description of homogeneous leukoplakia, the principal risk factor for malignant transformation, the presenting symptoms of oral submucous fibrosis, and the investigation required for definitive diagnosis.

The third part contained five items on self-reported clinical experience and perceptions: whether the respondent had ever detected a lesion suspicious for oral cancer, ever referred such a patient to a specialist, ever performed a biopsy of the oral mucosa, experienced difficulty in identifying OPMDs during clinical examination, and whether they considered a specialist in oral medicine or oral pathology to be the appropriate clinician to detect OPMDs. The full instrument is provided in Appendix A, and a supplementary table records the source instruments, the adaptation made and the rationale for each designated response (Table S1).

Content validity was assessed qualitatively by three faculty members in oral medicine and oral and maxillofacial diagnostic sciences. All items were reviewed for relevance, clarity, and freedom from ambiguity; the reviewers discussed wording and response options and reached consensus before pilot administration. No item was removed. The review was qualitative and no numerical item-level relevance ratings were collected at the time, so a content validity index cannot be computed retrospectively. The instrument was then pilot tested on 30 participants from the target population, who were excluded from the analytical sample. Pilot testing focused on clarity, comprehensibility, response-option usability and completion flow. Minor changes to wording and layout followed and no change was made to the intended constructs; an itemised record of the individual modifications was not retained.

### 2.5. Scoring and Outcome Definition

The primary outcome was a reduced seven-item knowledge score. Five items (B2, B3, B6, B10, and B12) were excluded because their wording or single-best-answer formulation did not permit sufficiently unambiguous interpretation. The retained items were B1, B4, B5, B7, B8, B9, and B11; each scored 1 for the designated response and 0 otherwise, giving a score from 0 to 7. The primary score was calculated only for respondents with non-missing responses to all seven retained items (n = 921); missing responses were not scored as incorrect or imputed. The full 12-item score (0–12) was examined as a sensitivity analysis among respondents with complete data for all 12 knowledge items (n = 920). A further six-item sensitivity score excluding B7 was calculated among respondents with complete responses for B1, B4, B5, B8, B9, and B11 (n = 921). The reduced score was not prespecified; it was defined after the initial analysis, in response to peer review, and all analyses based on it are therefore post hoc. It is a pragmatic summary of retained items and has not been validated as a psychometric scale. Internal consistency was quantified using the Kuder–Richardson 20 coefficient (equivalent to Cronbach’s alpha for dichotomous items). Item B7 was retained because it addresses relative lesion risk, but its wording does not distinguish per-lesion malignant risk from population-attributable burden; this ambiguity is explicitly acknowledged in the interpretation.

The five clinical experience and perception items were analysed separately. For C1–C3, ‘do not remember’ was retained as a distinct category in all descriptive summaries and was never recoded as ‘no’. For item-level significance testing, the response displayed in each table was compared with all other recorded responses combined, so that ‘do not remember’ contributed to the comparison category rather than being modelled as a third outcome level. These items describe lifetime self-reported experience and therefore depend on cumulative clinical opportunity as well as competence. No composite clinical-experience score was created.

### 2.6. Bias

Several potential sources of bias were identified, and the following measures were taken to address them. Selection bias was possible because recruitment used non-probability convenience sampling and voluntary participation. To broaden coverage within this sampling framework, the survey link was circulated throughout the 12-month recruitment period through institutional networks in three cities and was directed to all eligible participant groups, namely, clinical-year students, dental interns, and general dental practitioners. To reduce information bias, the questionnaire was adapted from previously published instruments, reviewed for content validity by three faculty members and pilot tested before use (Section 2.4). The designated responses were not shown to participants, and an ‘I do not know’ option was offered for all seven knowledge items retained in the primary score to discourage guessing. Five knowledge items with wording or single-best-answer limitations were excluded from the primary score (Section 2.5).

To reduce social-desirability bias, participation was voluntary, the confidentiality of responses was explained on the introductory page, and no direct identifiers were retained in the analytical dataset. To reduce recall bias in the clinical-experience items, a ‘do not remember’ option was provided and analysed as a separate category rather than recoded as ‘no’. Duplicate submissions were addressed by requiring respondent sign-in and screening for duplicates during data cleaning (Section 2.2), and pilot-test participants were excluded from the analytical sample. Measured potential confounding was addressed by adjustment for sex, age, qualification, and institutional affiliation in a multivariable model, and the robustness of the findings was examined in sensitivity analyses (Section 2.7); residual confounding from unmeasured variables could not be excluded. Residual bias from self-selection and self-report also could not be excluded, and these issues are considered further in Section 4.6.

### 2.7. Statistical Analysis

Analyses were performed using Python 3.13.5 with SciPy 1.17.0 and statsmodels 0.14.6. Categorical variables were summarised as frequencies and percentages using the number of available responses to each item as the denominator. Correct-response proportions for the seven retained knowledge items and the response shown for each clinical-experience item were compared across participant characteristics using Pearson chi-square tests (with Yates’ continuity correction for two-group comparisons) after excluding blank responses for the item being tested. To address multiple item-level comparisons, Benjamini–Hochberg false-discovery-rate (FDR) correction was applied across all comparisons within each table family; FDR-adjusted *p*-values are reported. ‘Do not remember’ responses were retained as substantive response categories and were not treated as ‘no’. Composite knowledge scores were analysed by complete case without imputation.

Because the reduced knowledge score was bounded and non-normal, unadjusted two-group comparisons used the Mann–Whitney U test and the three-category age comparison used the Kruskal–Wallis test. Multivariable linear regression included sex, age (20–25 years as reference, with 26–30 and >30-year indicators), qualification (trainee versus GDP), and institutional affiliation. HC3 heteroskedasticity-robust standard errors and 95% confidence intervals are reported. Model fit is summarised by R^2^. Residual diagnostics showed modest tail departure from normality (skewness −0.16; excess kurtosis 0.79); the correlation between fitted values and absolute residuals was 0.13, indicating only modest residual-scale patterning after use of HC3 standard errors. A Poisson regression with HC3 robust standard errors was fitted as an alternative count-model specification. A Poisson model does not impose the upper limit of the score; it tests whether the findings depend on the linear specification rather than providing a model that respects the bounds of the scale. Its exponentiated coefficients are ratios of expected scores. Sensitivity analyses comprised the full 12-item score, a 6-item score excluding B7, and a qualification analysis restricted to the 673 respondents whose recorded status was explicitly last-year student, intern, or GDP; a student-versus-intern comparison was also performed among the 472 respondents identifying one of those two trainee stages. Benjamini–Hochberg correction addresses multiplicity but not dependence between respondents recruited from the same institution. Institution-level identifiers were unavailable; so, cluster-robust standard errors could not be calculated; if responses are correlated within institutions, the standard errors of all coefficients and of all item-level comparisons may be underestimated, and the reported model R^2^ may be overly optimistic (inflated). A two-sided *p* < 0.05 was considered statistically significant.

### 2.8. Ethical Approval

The study was conducted in accordance with the Declaration of Helsinki and approved by the Research Ethics Committee of Riyadh Elm University (protocol code SPR/2022/102/761/721; approved 17 July 2022). The purpose of the research, the voluntary nature of participation, and the confidentiality of responses were explained on the introductory page of the questionnaire, and electronic informed consent was obtained from every participant before access to the items was granted. No direct identifiers were retained in the analytical dataset; sign-in information available during data cleaning was used only to screen for duplicate submissions.

## 3. Results

### 3.1. Characteristics of the Sample

**Figure 1:**
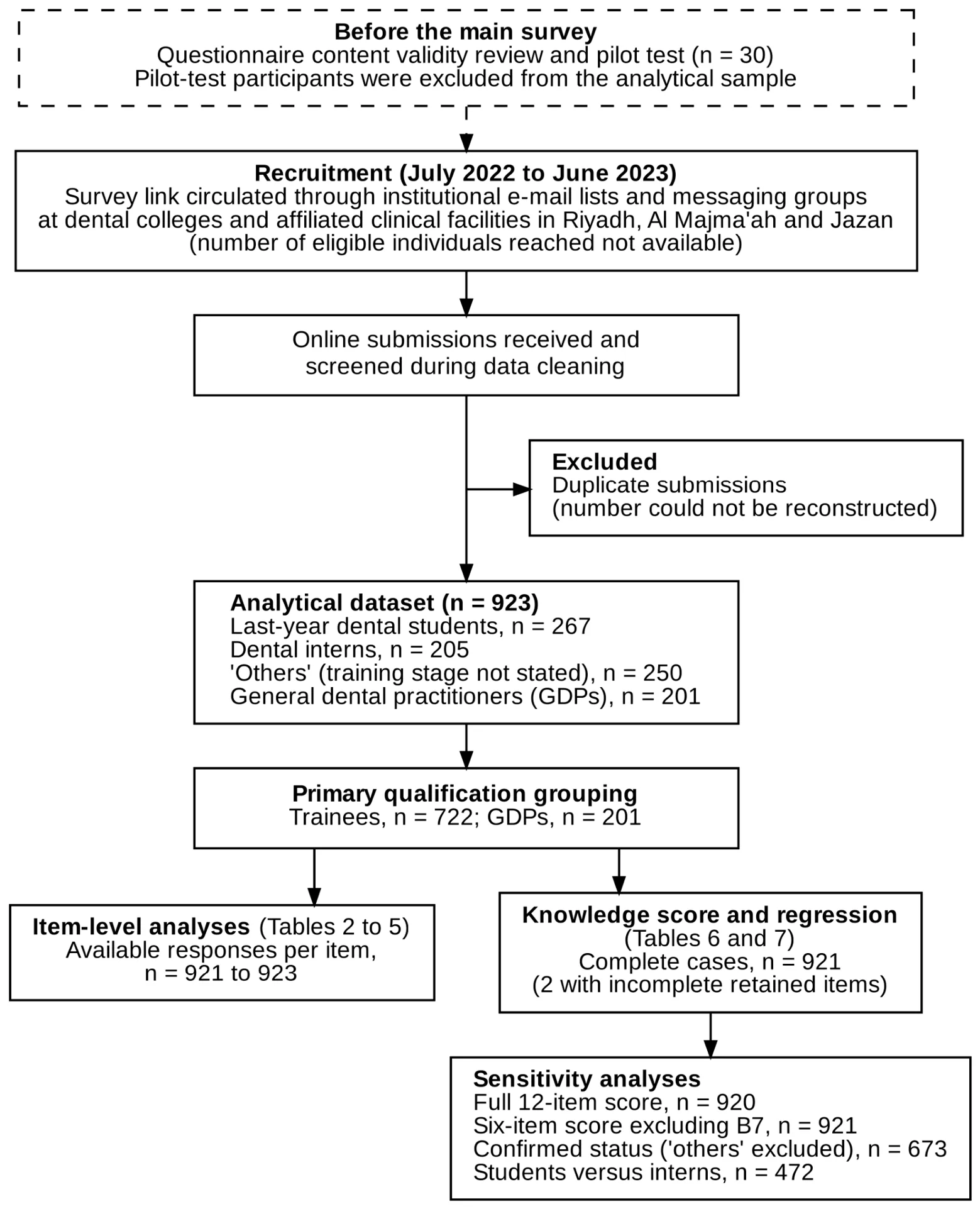
Flow diagram of participant recruitment, exclusions, and inclusion in the analyses. The pilot test (dashed box) was conducted before the main survey. The number of individuals reached through the institutional networks and the number of duplicate submissions removed during data cleaning could not be determined. GDP, general dental practitioner.

The flow of participants through recruitment, data cleaning, and analysis is shown in Figure 1. A total of 923 records were included; 920 (99.7%) were complete across all analysed fields. Slightly more than half were male (476, 51.6%). Age was recorded in the original three categories: 643 (69.7%) were 20–25 years, 172 (18.6%) were 26–30 years, and 108 (11.7%) were older than 30 years. The sample comprised 267 final-year dental students (28.9%), 205 dental interns (22.2%), 250 respondents selecting ‘others’ (27.1%), and 201 general dental practitioners (21.8%). For the primary qualification analyses, the first three categories were grouped as trainees (n = 722). Four in five respondents (738, 80.0%) reported government-institution affiliation (Table 1). Isolated item-level missing values are described in Section 3.2 and Section 3.3 and summarised in Table S6; missing values were handled as specified in Section 2.7.

### 3.2. Knowledge of Oral Potentially Malignant Disorders

Responses to the seven knowledge items retained in the primary score are shown in Table 2 by sex and age group and in Table 3 by qualification and institutional affiliation. The five excluded items (B2, B3, B6, B10, and B12) are reproduced in Appendix A for transparency but were not used in the primary score or subgroup knowledge tables because of their interpretive limitations.

Performance varied substantially across retained items. Leukoplakia was identified as the most common OPMD by 653 respondents (70.7%), and 828 (89.7%) selected ‘white lesion’ for the item on use of the term leukoplakia. Only 333 (36.1%) selected erythroplakia as the lesion most likely to show dysplasia or carcinoma; this result is interpreted cautiously because the question does not distinguish per-lesion malignant risk from population-attributable burden. Recognition of the early symptoms of oral submucous fibrosis was lowest, at 157 respondents (17.0%).

Item-level comparisons are accompanied by FDR-adjusted *p*-values (q-values). Almost all sex, age, and institutional-affiliation comparisons for the retained knowledge items remained statistically significant after correction. The exceptions were the qualification comparisons for erythroplakia (q = 0.162), erosive lichen planus (q = 0.519), and homogeneous leukoplakia (q = 0.244), for which trainees and general dental practitioners did not differ significantly. One response was missing for each of B7, B8, and B9 and two were missing for B11; percentages and tests for these items used the available responses.

### 3.3. Self-Reported Clinical Experience and Perceptions

Responses to the five clinical experience and perception items are presented in Table 4 and Table 5. For suspicious-lesion detection, among 922 available responses, 309 (33.5%) answered yes, 548 (59.4%) no, and 65 (7.0%) ‘do not remember’; one value was missing. For referral, among 922 available responses, 409 (44.4%) answered yes, 462 (50.1%) no, and 51 (5.5%) ‘do not remember’; one value was missing. For oral mucosal biopsy, all 923 records were observed; 147 (15.9%) answered yes, 728 (78.9%) no, and 48 (5.2%) ‘do not remember’. C4 contained one missing value and C5 contained two. ‘Do not remember’ responses were retained as separate categories and were not counted as absence of experience. Percentages and item-level tests used available responses for the relevant item.

Subgroup differences in the displayed clinical responses were tested with FDR correction (Table 4 and Table 5). All comparisons remained statistically significant after correction except the sex comparison for difficulty in identifying OPMDs (q = 0.965) and the institutional-affiliation comparison for biopsy experience (q = 0.491). General dental practitioners reported lower frequencies of previous suspicious-lesion detection (8.5% versus 40.5% among available responses) and referral (15.4% versus 52.4%) than trainees, while 95.5% of GDPs reported no previous biopsy. Because these questions concern lifetime experience, the direction of these contrasts is contrary to what cumulative clinical opportunity alone would predict. They therefore should not be interpreted as direct differences in competence or actual clinical activity and are considered likely to be influenced by differential interpretation of terms such as ‘detection’ and ‘referral’, supervision in teaching clinics, and unmeasured clinical exposure.

### 3.4. Knowledge Score

Among the 921 respondents with complete data for all seven retained items, the mean reduced knowledge score was 3.93 ± 1.82 of a possible 7 (56.1% of retained items correct) (Table 6). KR-20 was 0.68 for the seven-item score, indicating modest internal consistency across a deliberately broad set of knowledge domains; among 920 respondents complete on all 12 knowledge items, KR-20 was 0.74 and the mean full score was 5.99 ± 2.81. Reduced-score distributions differed by sex, age, qualification, and institutional affiliation (all omnibus or two-group *p* < 0.001). In a six-item sensitivity analysis excluding B7 (n = 921), the mean was 3.57 ± 1.52, KR-20 was 0.60, and every adjusted covariate retained the same direction and statistical significance as in the seven-item model (R^2^ = 0.582). In the confirmed-status sensitivity analysis, restricting analyses to the 673 respondents with explicitly identified student, intern, or GDP status markedly attenuated the unadjusted qualification contrast; identified trainees averaged 4.60 ± 1.25 versus 4.82 ± 1.30 for GDPs (*p* = 0.013). In the corresponding adjusted model, GDP status was associated with a 0.64-point higher score (95% CI 0.22 to 1.06; *p* = 0.003). In that model, the associations with male sex (B = 0.17; *p* = 0.394), age over 30 years (B = −0.84; *p* = 0.237), and private affiliation (B = −0.47; *p* = 0.102) were no longer statistically significant, whereas age 26–30 years remained associated with a lower score (B = −0.89; *p* < 0.001); R^2^ was 0.070 (Table S4). Among the 472 respondents who identified as either final-year student or intern, interns scored 5.42 ± 1.11 versus 3.96 ± 0.95 for students (*p* < 0.001). Although the mean seven-item score declined across the three age categories, the individual knowledge items did not show a uniform monotonic age gradient; several item-level proportions peaked in the 26–30-year group. The age findings therefore reflect heterogeneous item-specific patterns and the composition of this cross-sectional convenience sample rather than a consistent age-related progression.

Respondents affiliated with government institutions had a higher unadjusted reduced score than those with private affiliation (4.46 ± 1.38 versus 1.79 ± 1.80). However, institutional identity was not recorded and affiliation could not be disentangled from study site, curriculum, region, or case mix. This comparison is therefore retained only as an exploratory within-sample observation and is not interpreted as a government-versus-private sector effect.

### 3.5. Characteristics Associated with the Knowledge Score

In the multivariable complete-case model for the seven-item score (n = 921; Table 7), male sex was associated with a 0.95-point higher score (95% CI 0.65 to 1.24), age 26–30 years with a 0.72-point lower score (95% CI −1.01 to −0.42), age >30 years with a 1.78-point lower score (95% CI −2.33 to −1.22), GDP status with a 1.50-point higher score (95% CI 1.21 to 1.79), and private-institution affiliation with a 1.12-point lower score (95% CI −1.49 to −0.75), all *p* < 0.001 using HC3 robust standard errors. The model R^2^ was 0.539. An alternative Poisson specification produced the same direction and statistical significance for all covariates: ratios of expected scores were 1.34 (95% CI 1.23–1.47) for male sex, 0.86 (0.79–0.93) for age 26–30 years, 0.43 (0.30–0.61) for age >30 years, 1.53 (1.40–1.67) for GDP status, and 0.66 (0.57–0.77) for private affiliation (all *p* < 0.001). On the same 920 respondents with complete 12-item data, the 7-item and full 12-item models retained the same direction of association for every covariate (R^2^ = 0.537 and 0.629, respectively); coefficients are reported side by side in Table S2 and are not directly comparable in magnitude because the score ranges differ. The six-item and confirmed-status sensitivity analyses are reported in Tables S3 and S4, and Poisson estimates in Table S5. In the confirmed-status sensitivity analysis (n = 673; Table S4), the GDP association was retained but reduced (B = 0.64 versus 1.50 in the primary model), and age 26–30 years remained associated with a lower score (B = −0.89; *p* < 0.001); in contrast, the associations with male sex (*p* = 0.394), age >30 years (*p* = 0.237) and private affiliation (*p* = 0.102) were no longer statistically significant, and R^2^ was 0.070 compared with 0.539 in the primary model. The primary-model associations for sex, age over 30 years, and institutional affiliation therefore depended materially on inclusion of respondents recorded as ‘others’. These estimates describe associations within this convenience sample and do not establish causal or sector-level effects.

## 4. Discussion

### 4.1. Principal Findings

In this convenience sample of 923 respondents recruited through selected institutional networks, 921 respondents with complete retained-item data had a seven-item knowledge score averaging 3.93 of 7 (56.1%). Excluding five items with important wording or single-best-answer limitations did not eliminate substantive knowledge gaps, although the reduced score showed only modest internal consistency (KR-20 = 0.68) and should be interpreted as a broad index rather than a unidimensional psychometric scale. A six-item sensitivity analysis excluding the wording-sensitive B7 item retained the direction and statistical significance of all adjusted associations, although internal consistency decreased to KR-20 = 0.60. Self-reported lesion detection, referral, and biopsy were also limited, but these lifetime measures cannot be interpreted as standardised measures of competence.

The analysis separates knowledge from self-reported experience and also separates ‘do not remember’ from negative experience responses. This distinction matters because cumulative clinical opportunity, supervision, and interpretation of survey terminology can affect the experience items independently of knowledge.

Knowledge and self-reported clinical activity were not concordant. In the primary classification, general dental practitioners recorded a higher mean knowledge score than the full trainee group (4.82 versus 3.68 of 7) yet reported markedly less lifetime lesion detection (8.5% versus 40.5% among available responses) and referral (15.4% versus 52.4%). The knowledge-score contrast was substantially attenuated when the 250 respondents recorded as ‘others’ were excluded; GDPs averaged 4.82 versus 4.60 among the 472 identified students and interns (*p* = 0.013), and the adjusted GDP coefficient fell to 0.64 points. This sensitivity analysis reinforces that the primary qualification contrast is strongly dependent on how the unverified ‘others’ category is classified. These are comparisons of group averages; they do not establish whether individual respondents with higher knowledge scores were more or less likely to report clinical experiences, and no individual-level knowledge–experience association was prespecified. The experience items recorded whether an event had ever occurred rather than how often it occurred per patient examined, and the same wording may have been read differently by respondents working under supervision and by those in independent practice.

### 4.2. Recognition of Lesions and Stratification of Risk

Leukoplakia was well recognised, both as a term and as the commonest OPMD, by 89.7% and 70.7% of respondents, respectively. This exceeds the 62.4% reported by Hassona et al. among Jordanian participants [26] and is comparable with figures of approximately 68% observed in both dentists and students in a recent Portuguese study [27]. This item asked only whether the term denotes a white lesion, however, and a correct response does not demonstrate command of the contemporary definition, which specifies a predominantly white plaque of questionable risk once other recognised disorders carrying no increased cancer risk have been excluded [6]. The high accuracy on this item should not be read as evidence of thorough understanding of leukoplakia.

Erythroplakia, whose clinicopathological features are described across reviews and case series [28,29], remains clinically important because individual erythroplakic lesions carry a high probability of dysplasia or carcinoma and a substantial malignant-transformation risk [8,30]. In the present survey, 36.1% selected erythroplakia for the item asking which lesion was ‘much more likely to show dysplasia or carcinoma’. That result should not be treated as a pure knowledge deficit, however, because the wording did not distinguish per-lesion risk from the absolute number of dysplastic or malignant cases arising in a population, which is also influenced by lesion prevalence. This proportion is higher than the 11.4% reported among dentists in an Indonesian study [31], although differences in questionnaire wording and populations limit direct comparison. The item was retained in the reduced score because it addresses relative lesion risk, but its ambiguous wording is explicitly acknowledged and no educational recommendation rests on this item alone.

For oral lichen planus, 64.0% of respondents identified the erosive subtype as carrying the greatest malignant potential, consistent with systematic-review evidence of greater malignant-transformation risk in erosive and atrophic forms than in reticular disease [32].

The item on whether oral epithelial dysplasia ‘goes away’ was one of five items excluded from the primary score because its dichotomous framing does not reflect the possibility of persistence, progression, or regression. Likewise, the items regarding ambiguous diagnostic interval, erythroplakia site, malignant transformation risk, and broad histopathology were excluded from the reduced score. This approach preserves transparency by retaining the full questionnaire in Appendix A while removing from the main knowledge outcome the five items whose wording could not be defended. It does not make the reduced score free of interpretive difficulty, because item B7 was retained despite the wording ambiguity described above.

Recognition of oral submucous fibrosis was the weakest area, with 17.0% identifying its characteristic early symptoms. This may partly reflect the lower prevalence of areca-nut chewing in Saudi Arabia relative to South and Southeast Asia and consequently more limited clinical exposure to the condition. Nonetheless, population mobility from regions where areca-nut use is endemic means that clinicians may still encounter the condition, so it represents a clinically relevant knowledge gap rather than a purely academic one.

### 4.3. Lesion Detection, Referral and Biopsy

In this sample, 59.4% of respondents answered that they had not previously detected a lesion suspicious for oral cancer, while 7.0% selected ‘do not remember’; these response categories were analysed separately. This may reflect limited clinical exposure, failure of recognition, or both, and the present survey cannot distinguish between these possibilities. The item asks about lifetime experience, so that it is influenced by years in practice and patient volume, neither of which were recorded; for students in particular, a negative response may be entirely expected. Golburean et al. reported a closely comparable figure of 60.4% among dentists in Moldova, Belarus and Armenia [33], whereas a Canadian study at a single well-resourced academic centre found substantially fewer respondents reporting no such encounter [34], a contrast plausibly attributable to differences in clinical exposure and case mix.

General dental practitioners reported lower frequencies of previous lesion detection and referral than trainees, a direction that cumulative clinical opportunity alone would not predict. Because the items ask whether an event has ever occurred, and because years in practice, patient volume, prior oral medicine rotations, and supervised teaching-clinic exposure were not measured, the contrast cannot be interpreted as a true difference in diagnostic activity or competence. Differential interpretation is a particularly plausible source of bias; trainees may count escalation to a supervising clinician as ‘referral’ or use a lower threshold for labelling a lesion as suspicious, whereas practitioners may interpret both terms more narrowly. These explanations are speculative and could not be tested with the present data. The finding is therefore treated as hypothesis-generating, and most plausibly as a measurement artefact, rather than as a substantive comparison of trainee and practitioner performance.

Referral showed a similar pattern; 50.1% reported no previous referral and 5.5% selected ‘do not remember’, while 73.3% agreed that an oral medicine or oral pathology specialist is the appropriate clinician to detect these lesions. Two readings are available and the data cannot separate them. Respondents may recognise the limits of their own competence without having an established referral pathway through which to act; alternatively, they may regard lesion detection as primarily a specialist responsibility, which would reduce the attention given to mucosal examination in their own practice. This distinction matters for intervention design and merits direct investigation.

Biopsy experience was limited, with 78.9% reporting no previous oral mucosal biopsy and 5.2% selecting ‘do not remember’. Limited biopsy experience among general dental practitioners has also been reported elsewhere [35], and diagnostic accuracy for dysplastic OPMDs has been reported to be higher among specialists than among general clinicians [36]. We do not contend that every general dentist should routinely biopsy OPMDs. The American Dental Association guidelines for potentially malignant oral lesions recommend biopsy or referral to a specialist when a lesion is clinically suspicious, supporting a role for general dentists in recognition and timely diagnostic escalation rather than an expectation that all clinicians perform biopsy themselves [37]. Limited biopsy experience may therefore reflect training, confidence, referral arrangements, local scope-of-practice expectations, or case mix; these mechanisms were not measured.

### 4.4. Variation by Participant Characteristics and Institutional Setting

Participant characteristics other than institutional affiliation were also associated with the reduced knowledge score in the primary model. Scores were lower by 0.72 points at 26–30 years and by 1.78 points above 30 years relative to the youngest category; GDP status was associated with a 1.50-point higher score and male sex with a 0.95-point higher score. However, the confirmed-status sensitivity analysis reduced the adjusted GDP association to 0.64 points (95% CI 0.22 to 1.06; *p* = 0.003), showing that the qualification result is sensitive to classification of respondents recorded as ‘others’. The same analysis removed the statistical significance of the associations with male sex and with age over 30 years (B = −0.84; *p* = 0.237), indicating that these associations also depended largely on the inclusion of respondents recorded as ‘others’. Age and qualification therefore should not be read as simple proxies for cumulative clinical experience in this sample. The steep decline in the mean score across the three age groups (Table 6) should likewise not be read as an effect of age itself. The oldest group scored lowest even though general dental practitioners, who might be expected to be older on average, scored highest, and several individual items peaked in the 26–30-year group (Table 2). Because the association in the over-30-years group lost statistical significance when respondents recorded as ‘others’ were excluded, the age pattern most plausibly reflects differences between age groups in qualification mix, training stage, institutional and site composition, and clinical exposure rather than a consistent age-related progression; the cross-sectional convenience design does not support a causal interpretation of age. Because sex, age, qualification, and institutional affiliation were unevenly distributed across the participating networks, and because year of graduation, curriculum content, and previous continuing education were not recorded, these associations describe the composition of this convenience sample rather than characteristics of the wider dental workforce.

Institutional affiliation remained associated with the reduced knowledge score, at −1.12 points on the 0 to 7 scale for private versus government affiliation, and it was not the largest adjusted association in the model. When respondents recorded as ‘others’ were excluded, the association was attenuated to −0.47 points and was no longer statistically significant (*p* = 0.102; Table S4). The 7- and 12-item scores have different ranges; so, the corresponding coefficients cannot be compared directly in magnitude. More importantly, institutional identity was not recorded, and affiliation thus cannot be separated from study site, region, curriculum, participant composition, or case mix. The observation is therefore exploratory and should not be interpreted as evidence of a private-sector effect.

Because respondents were recruited from a limited number of institutions, observations from the same institution may also be correlated through shared curriculum, clinical environment, and referral systems. Institution-level identifiers were unavailable, so that neither centre-specific estimates nor cluster-robust standard errors could be calculated. The HC3 robust errors used in the regression address heteroskedasticity but do not correct within-institution dependence. Accordingly, within-institution correlation could underestimate standard errors for all regression coefficients and could make the reported R^2^ appear overly optimistic.

For trainees, affiliation denotes the training institution rather than a clinical workplace. This further limits any sector-based interpretation. The appropriate use of the institutional result is therefore to motivate future studies that record institution identity and recruit enough centres within each sector to separate sector, site, curriculum, and region.

### 4.5. Implications for Continuing Education

The coexistence of knowledge gaps with limited self-reported lesion detection, referral, and biopsy is consistent with a need to assess clinically applied competencies in addition to factual knowledge. The present study did not compare educational interventions or establish that competency-based education is superior to didactic teaching; so, the following points are framed as areas for future educational development and evaluation rather than as tested effects.

Potential priorities include risk-stratified lesion recognition, an operational threshold for assessment of persistent unexplained lesions, clear referral pathways with diagnostic feedback, and supervised biopsy training where this falls within the intended scope of practice. These priorities arise from the domains in which uncertainty or limited exposure was observed; their effectiveness would need to be tested prospectively rather than inferred from this cross-sectional survey.

There is supporting evidence that educational interventions can improve knowledge of oral cancer, although evidence for translation into clinical practice is less direct. A structured educational intervention among dentists in northern Germany produced measurable gains in oral cancer knowledge [38], and a more recent multicentre randomised trial in Colombia found that a massive open online course improved knowledge, attitudes, and practice scores among dentists and dental students relative to a printed leaflet and to no training [39]. A systematic review and meta-analysis of training programmes for frontline health workers reported improvements in knowledge scores that did not reach statistical significance, with substantial heterogeneity between studies, while supporting the feasibility of strengthening detection capacity at the primary care level [40]. A recent scoping review of how oral cancer is taught identified wide variation in the mode of delivery of oral cancer education among practitioners and students, and persistent gaps in existing approaches [41]. Direct clinical observation has been used to identify the behavioural factors that determine whether early detection occurs [42], and a Delphi consensus of general dentists and oral medicine experts converged on the need for continuing education in this area [43]. Regional evidence from Jazan [44], Yemen [45,46], the United Arab Emirates [47], and elsewhere in Saudi Arabia [48] points in the same direction, and the most recent Saudi survey reported a clear discrepancy between oral cancer knowledge and preventive practice among dental students and professionals, concluding that targeted training was required [49].

### 4.6. Limitations

This study has several limitations. The most important concerns generalisability. Participants were recruited from dental colleges and affiliated clinical facilities in only three cities; thus, the findings cannot be generalised to the national dental workforce, to other regions of Saudi Arabia, or to dentists working outside the participating institutional networks.

This study was also subject to sampling bias. Non-probability convenience sampling was used and participation was voluntary, so that respondents with greater interest in oral medicine or greater confidence in their knowledge may have been more likely to take part, whereas those less engaged with the topic may be under-represented. The direction and magnitude of any resulting selection bias cannot be determined from the available data. Because the membership of the distribution networks was unknown, a response rate could not be calculated and the extent of non-response bias could not be estimated. The sample was also unbalanced, with most respondents aged 20 to 25 years (69.7%) and affiliated with government institutions (80.0%), which may have influenced both the overall estimates and the subgroup comparisons.

The sample size also limits for national inference and several subgroup analyses, and no a priori sample-size calculation was performed. Subgroups such as the 201 GDPs, the 185 respondents with private affiliation, and the 108 respondents older than 30 years were small, which means that estimates for these groups are less precise and the study may have lacked power to detect small differences in individual items within them.

Data on several important potential confounders were not collected. These include years since graduation and duration of clinical practice, patient volume, practice setting, previous oral medicine rotations or other clinical training in oral mucosal disease, previous continuing education on oral cancer, the country or institution of undergraduate training, and the specific institution attended. The associations reported in Table 7 may therefore be affected by residual confounding and should not be interpreted as independent or causal effects.

Other limitations should also be noted. The final analytical file contained isolated item-level missing values; analyses therefore used available responses for individual items and complete cases for composite scores, without imputation. The deidentified analytical file did not retain the sign-in metadata used during original cleaning, so the number of submissions excluded as duplicates cannot be reconstructed retrospectively. Clinical experience was self-reported, introducing recall and social-desirability bias, and the cross-sectional design supports association rather than causation. Because knowledge, experience, and participant characteristics were measured at a single time point, the temporal sequence of these relationships cannot be established.

Four additional limitations are central to subgroup interpretation. First, institutional affiliation is confounded with site and other unmeasured institutional characteristics, and institution-level identifiers were unavailable for centre-specific or cluster-robust analyses. Second, detection, referral, and biopsy are lifetime experiences shaped by years in practice and patient volume; the striking trainee–GDP contrasts are therefore vulnerable to differential item interpretation and unequal opportunity. Third, the trainee group included 250 respondents who selected ‘others’ and whose training stage could not be confirmed. In a sensitivity analysis restricted to the 673 respondents with explicit student, intern, or GDP status, the GDP-versus-trainee knowledge difference persisted but was markedly attenuated (4.82 versus 4.60; *p* = 0.013), and interns scored higher than final-year students among the 472 identified trainees. The primary qualification, sex, age, and institutional-affiliation findings therefore depend materially on classification of the ‘others’ category; in the restricted analysis, the associations with sex, age over 30 years, and affiliation were no longer statistically significant. Years since graduation, patient volume, prior oral medicine rotations, and previous continuing education were unavailable. Fourth, item-level subgroup analyses are numerous; FDR correction reduces but does not eliminate the risk of chance findings.

The questionnaire also has measurement limitations. Five knowledge items (B2, B3, B6, B10, and B12) were excluded from the primary score because their wording or single-best-answer structure was not sufficiently defensible. The reduced seven-item score had modest internal consistency (KR-20 = 0.68), consistent with a brief instrument sampling several domains rather than a single narrow construct. Item B7 remains imperfect because ‘more likely to show dysplasia or carcinoma’ can be interpreted as per-lesion risk or population burden; it is therefore interpreted cautiously. Because B7 was retained, the reduced score is not entirely free of items with acknowledged wording limitations. Content validity was also assessed qualitatively, without a content validity index. These limitations mean that the score is best regarded as a pragmatic summary of retained items rather than a fully validated psychometric scale. The reduced score, the false-discovery-rate correction, the robust standard errors, and the count-model sensitivity analysis were all defined after the initial analysis rather than prespecified, and the results based on them are correspondingly post hoc.

Finally, data were collected between July 2022 and June 2023 and reflect knowledge, self-reported clinical experience, and perceptions during that period; curricular and continuing education provision may have developed since. Longitudinal or interventional designs and studies incorporating direct clinical observation or standardised patients would be better placed to establish whether targeted continuing education changes clinical practice rather than knowledge alone.

## 5. Conclusions

This cross-sectional study of dental trainees and general dental practitioners recruited through selected institutional networks in Saudi Arabia found a mean seven-item OPMD knowledge score of 3.93 ± 1.82 of 7, together with limited self-reported lifetime experience of suspicious-lesion detection, referral, and oral mucosal biopsy.

In the multivariable model (Table 7), male sex and GDP status were associated with higher knowledge scores, whereas age 26 to 30 years, age over 30 years, and private-institution affiliation were associated with lower knowledge scores. In a sensitivity analysis excluding respondents whose training stage was not stated, only the associations with GDP status and age 26 to 30 years remained statistically significant. Because all variables were measured at a single time point in a non-probability sample, these findings show associations only. They do not establish causality or the direction of the relationships and cannot be generalised to all dentists in Saudi Arabia.

Longitudinal studies using probability sampling, larger subgroups, and data on potential confounders, such as years in practice and previous continuing education, are needed to evaluate the robustness of these associations.

## Figures and Tables

**Table 1:** Sociodemographic and professional characteristics of the study sample (*n* = 923).

Characteristic	n	%
Sex		
Male	476	51.6
Female	447	48.4
Age group		
20–25 years	643	69.7
26–30 years	172	18.6
>30 years	108	11.7
Recorded level		
Final-year dental student	267	28.9
Dental intern	205	22.2
Other/unspecified (‘others’)	250	27.1
General dental practitioner	201	21.8
Institutional affiliation		
Government	738	80.0
Private	185	20.0

**Table 2:** Responses to the knowledge items for the whole sample and by sex and age group.

Item (Designated Response)	All	Male	Female	q (Sex)	20–25	26–30	>30	q (Age)
OPMDs always progress to malignancy (no)	548 (59.4)	218 (45.8)	330 (73.8)	<0.001	365 (56.8)	152 (88.4)	31 (28.7)	<0.001
Most common OPMD (leukoplakia)	653 (70.7)	442 (92.9)	211 (47.2)	<0.001	594 (92.4)	42 (24.4)	17 (15.7)	<0.001
Correct use of the term leukoplakia (a white lesion)	828 (89.7)	467 (98.1)	361 (80.8)	<0.001	624 (97.0)	169 (98.3)	35 (32.4)	<0.001
Lesion most likely to show dysplasia or carcinoma (erythroplakia)	333 (36.1)	236 (49.6)	97 (21.7)	<0.001	305 (47.4)	21 (12.2)	7 (6.5)	<0.001
Subtype of lichen planus with greatest malignant potential (erosive)	590 (64.0)	431 (90.5)	159 (35.7)	<0.001	544 (84.6)	33 (19.2)	13 (12.1)	<0.001
Flat, thin, uniformly white lesion with shallow surface cracks (homogeneous leukoplakia)	509 (55.2)	358 (75.2)	151 (33.9)	<0.001	469 (72.9)	26 (15.1)	14 (13.1)	<0.001
Burning sensation and intolerance of spicy food as presenting symptoms (oral submucous fibrosis)	157 (17.0)	25 (5.3)	132 (29.7)	<0.001	83 (12.9)	70 (40.7)	4 (3.8)	<0.001

Figures are n (%) giving the designated response, with percentages calculated from the available responses for each item and subgroup. Only the seven items retained in the primary score are shown. q-values are Benjamini–Hochberg FDR-adjusted *p*-values from Pearson chi-square tests; the FDR family for this table comprises all 14 *p*-values (seven knowledge items × two subgroup characteristics: sex and age), adjusted together within Table 2 only. OPMD, oral potentially malignant disorder.

**Table 3:** Responses to the knowledge items by qualification and institutional affiliation.

Item (Designated Response)	Trainee (n = 722)	GDP (n = 201)	q (Qualification)	Government (n = 738)	Private (n = 185)	q (Affiliation)
OPMDs always progress to malignancy (no)	367 (50.8)	181 (90.0)	<0.001	452 (61.2)	96 (51.9)	0.033
Most common OPMD (leukoplakia)	474 (65.7)	179 (89.1)	<0.001	621 (84.1)	32 (17.3)	<0.001
Correct use of the term leukoplakia (a white lesion)	630 (87.3)	198 (98.5)	<0.001	719 (97.4)	109 (58.9)	<0.001
Lesion most likely to show dysplasia or carcinoma (erythroplakia)	251 (34.8)	82 (40.8)	0.162	317 (43.0)	16 (8.7)	<0.001
Subtype of lichen planus with greatest malignant potential (erosive)	457 (63.4)	133 (66.2)	0.519	558 (75.6)	32 (17.4)	<0.001
Flat, thin, uniformly white lesion with shallow surface cracks (homogeneous leukoplakia)	390 (54.1)	119 (59.2)	0.244	479 (64.9)	30 (16.3)	<0.001
Burning sensation and intolerance of spicy food as presenting symptoms (oral submucous fibrosis)	81 (11.2)	76 (37.8)	<0.001	144 (19.5)	13 (7.1)	<0.001

GDP, general dental practitioner. Trainee includes all non-GDP recorded levels. Percentages use available item-level responses. q-values are Benjamini–Hochberg FDR-adjusted *p*-values from Pearson chi-square tests; the FDR family for this table comprises all 14 *p*-values (seven knowledge items × two subgroup characteristics: qualification and institutional affiliation), adjusted together within Table 3 only. Institutional-affiliation comparisons are exploratory because affiliation could not be separated from study site.

**Table 4:** Responses to the clinical experience and perception items for the whole sample and by sex and age group.

Item (Response Shown)	All	Male	Female	q (Sex)	20–25	26–30	>30	q (Age)
Has detected a lesion suspicious for oral cancer (yes)	309 (33.5)	278 (58.4)	31 (7.0)	<0.001	283 (44.0)	15 (8.7)	11 (10.3)	<0.001
Has referred a patient to a specialist (yes)	409 (44.4)	363 (76.3)	46 (10.3)	<0.001	366 (56.9)	27 (15.7)	16 (15.0)	<0.001
Has performed a biopsy of the oral mucosa (no)	728 (78.9)	341 (71.6)	387 (86.6)	<0.001	501 (77.9)	161 (93.6)	66 (61.1)	<0.001
Experiences difficulty in identifying OPMDs on examination (no)	327 (35.5)	168 (35.3)	159 (35.7)	0.965	306 (47.6)	15 (8.7)	6 (5.6)	<0.001
Considers an oral medicine or oral pathology specialist the appropriate clinician to detect OPMDs (yes)	675 (73.3)	440 (92.4)	235 (52.8)	<0.001	592 (92.1)	66 (38.4)	17 (16.0)	<0.001

Figures are n (%) giving the response shown in the first column, with percentages calculated from the available responses to each item. For C1–C3, ‘do not remember’ was retained as a separate descriptive category and was not recoded as ‘no’. The available denominators were 922 for C1, 922 for C2, and 923 for C3; blank responses were excluded from the relevant item-level test. q-values are Benjamini–Hochberg FDR-adjusted *p*-values from Pearson chi-square tests comparing the displayed response with all other recorded response options; the FDR family for this table comprises all 10 *p*-values (five items × two subgroup characteristics: sex and age), adjusted together within Table 4 only.

**Table 5:** Responses to the clinical experience and perception items by qualification and institutional affiliation.

Item (Response Shown)	Trainee (n = 722)	GDP (n = 201)	q (Qualification)	Government (n = 738)	Private (n = 185)	q (Affiliation)
Has detected a lesion suspicious for oral cancer (yes)	292 (40.5)	17 (8.5)	<0.001	297 (40.2)	12 (6.5)	<0.001
Has referred a patient to a specialist (yes)	378 (52.4)	31 (15.4)	<0.001	383 (51.9)	26 (14.1)	<0.001
Has performed a biopsy of the oral mucosa (no)	536 (74.2)	192 (95.5)	<0.001	586 (79.4)	142 (76.8)	0.491
Experiences difficulty in identifying OPMDs on examination (no)	193 (26.8)	134 (66.7)	<0.001	309 (41.9)	18 (9.8)	<0.001
Considers an oral medicine or oral pathology specialist the appropriate clinician to detect OPMDs (yes)	491 (68.2)	184 (91.5)	<0.001	631 (85.5)	44 (24.0)	<0.001

GDP, general dental practitioner. Trainee includes all non-GDP recorded levels. Percentages use available item-level responses. The items reflect lifetime self-reported experience, not standardised opportunities or observed competence; GDP-versus-trainee contrasts may therefore be strongly influenced by different interpretation of the questions and by unmeasured duration and volume of clinical exposure. For C1–C3, the available denominators were 922, 922, and 923, respectively. q-values are Benjamini–Hochberg FDR-adjusted *p*-values; the FDR family for this table comprises all 10 *p*-values (five items × two subgroup characteristics: qualification and institutional affiliation), adjusted together within Table 5 only.

**Table 6:** Knowledge score for the whole sample and by participant characteristics.

Variable	Category	Mean ± SD	*p*
Whole sample	Complete cases (n = 921)	3.93 ± 1.82	
Sex	Male (n = 476)	4.57 ± 1.30	<0.001
	Female (n = 445)	3.24 ± 2.03	
Age group	20–25 years (n = 643)	4.64 ± 1.32	<0.001
	26–30 years (n = 172)	2.98 ± 1.21	
	>30 years (n = 106)	1.14 ± 1.85	
Qualification	Trainee (n = 720)	3.68 ± 1.86	<0.001
	GDP (n = 201)	4.82 ± 1.30	
Institutional affiliation	Government (n = 738)	4.46 ± 1.38	<0.001
	Private (n = 183)	1.79 ± 1.80	

*p*-values are from the Mann–Whitney U test for two-category variables and the Kruskal–Wallis test for age group. Possible reduced-score range 0–7. Composite-score analyses are complete case (n = 921 overall). The two respondents excluded from Table 6 because of incomplete retained-item data were both female, >30 years old, classified as trainees, and privately affiliated; this explains why those four subgroup counts are each two lower than the corresponding counts in Table 1. SD, standard deviation. Institutional-affiliation results are exploratory because affiliation could not be separated from site.

**Table 7:** Multivariable linear regression model for participant characteristics associated with the knowledge score.

Characteristic	B	Robust SE	95% CI Lower	95% CI Upper	*p*
Male sex (vs. female)	0.95	0.15	0.65	1.24	<0.001
Age 26–30 years (vs. 20–25)	−0.72	0.15	−1.01	−0.42	<0.001
Age > 30 years (vs. 20–25)	−1.78	0.28	−2.33	−1.22	<0.001
GDP (vs. trainee)	1.50	0.15	1.21	1.79	<0.001
Private affiliation (vs. government)	−1.12	0.19	−1.49	−0.75	<0.001

Dependent variable: reduced knowledge score (0–7), analysed among 921 complete cases. B, unstandardised coefficient; CI, confidence interval; GDP, general dental practitioner; SE, HC3 robust standard error. Reference categories are female sex, age 20–25 years, trainee status, and government affiliation. R^2^ = 0.539. Institution-level clustering could not be modelled because institution identifiers were unavailable. If responses are correlated within institutions, the standard errors of all coefficients in this model, not only that for institutional affiliation, may be underestimated, and the reported R^2^ may be inflated. The institutional-affiliation coefficient is additionally exploratory because affiliation could not be separated from study site.

## Data Availability

The individual-level dataset is not publicly available because the participant information and consent specified that responses would be accessible only to the research team and reported in aggregate. The aggregate tabulations and regression output supporting the reported results are available from the corresponding author on reasonable request.

## Supplementary Materials

The following supporting information can be downloaded at: https://www.mdpi.com/article/10.3390/healthcare14203463/s1, Table S1: provenance and basis of each questionnaire item, showing the source instruments, adaptation and rationale for each designated response; Table S2: side-by-side seven-item and 12-item multivariable models on the same respondents; Table S3: six-item sensitivity analysis excluding B7; Table S4: qualification analyses restricted to respondents with explicitly identified status; Table S5: Poisson sensitivity model with 95% confidence intervals; Table S6: item-level missing values in the final analytical dataset; File S1: completed STROBE checklist; File S2: completed CHERRIES checklist; File S3: completed CROSS checklist.

## Abbreviations

The following abbreviations are used in this manuscript:

CI	Confidence interval
GDP	General dental practitioner
OPMD	Oral potentially malignant disorder
SD	Standard deviation
SE	Standard error
STROBE	Strengthening the Reporting of Observational Studies in Epidemiology

## Appendix A

### Study Questionnaire

The instrument administered to participants is reproduced below. Designated responses used in the original knowledge coding are shown in italics; these were not indicated to participants. The primary knowledge score excludes B2, B3, B6, B10, and B12 because of the interpretive limitations described in Section 2.5 and Section 4.6.

**Part A. Participant characteristics**

A1. Sex: male/female.
A2. Age group: 20–25 years/26–30 years/>30 years.
A3. Level: last-year dental student/dental intern/dental general practitioner/others.
A4. Place of study or work: government/private.

**Part B. Knowledge items**

B1. Will an oral potentially malignant disorder always progress to malignancy? yes/*no*/I do not know.
B2. Does oral dysplasia go away? yes/*no*/I do not know.
B3. How long is required to run a precise diagnostic procedure when there is no chance for an oral lesion to be cured? less than 1 week/less than 2 weeks/*more than 2 weeks*.
B4. What is the most common type of oral potentially malignant disorder? *leukoplakia*/erythroplakia/lichen planus/I do not know.
B5. The usage of the term leukoplakia: *white lesion*/red lesion/oral ulcer/I do not know.
B6. The most common site of oral erythroplakia: *tongue*/lip/hard palate/soft palate/I do not know.
B7. Much more likely to show dysplasia or carcinoma: leukoplakia/*erythroplakia*/lichen planus/oral submucous fibrosis/I do not know.
B8. Which type of lichen planus is more likely to be malignant: reticular LP/plaque-like LP/*erosive LP*/I do not know.
B9. Uniformly flat, thin, uniformly white in colour and showing shallow cracks of the surface keratin is: *homogenous leukoplakia*/non-homogeneous leukoplakia/oral submucous fibrosis/I do not know.
B10. Which of the following is considered the most important risk factor for malignant transformation: *location of lesion*/size of lesion/homogenous leukoplakia/non-homogeneous leukoplakia/I do not know.
B11. Symptoms such as a burning sensation and/or intolerance to spicy food are the most common symptoms in the initial phase of: leukoplakia/erythroplakia/lichen planus/*oral submucous fibrosis*/I do not know.
B12. Which of the following is necessary in order to reach a definitive diagnosis of oral lesions: clinical examination/radiographic examination/*histopathological examination*/all three have the same value.

**Part C. Clinical experience and perception items**

C1. Have you ever detected a suspicious lesion for oral cancer? yes/no/do not remember.
C2. Have you ever referred a patient to a specialist? yes/no/do not remember.
C3. Have you ever performed a biopsy of the oral mucosa? yes/no/do not remember.
C4. Do you experience difficulties in identifying OPMDs during the clinical examination? yes/no/not sure.
C5. Do you think that a specialist in oral medicine or oral pathology is the typical specialist to detect OPMDs? yes/no/not sure.
