# Supplementary material for: Knowledge and Self-Reported Clinical Experience of Oral Potentially Malignant Disorders Among Dental Trainees and General Dentists in Saudi Arabia: A Cross-Sectional Study
Source: Healthcare (Basel). 2026 Oct 15;14(20):3463. doi: 10.3390/healthcare14203463 (PMC13704271; doi:10.3390/healthcare14203463)
Supplement: Supplementary file 1 [file healthcare-14-03463-s001.zip › File S1 STROBE checklist.pdf]

File S1. Completed STROBE checklist

STROBE Statement: checklist of items for reports of cross-sectional studies

Manuscript ID: healthcare-4583004
Oral Potentially Malignant Disorders: Knowledge and Self-Reported Clinical Experience Among Dental Trainees and General Dentists in Saudi Arabia

Item descriptors are summarised. Locations refer to sections, tables and appendices of the revised manuscript.

Section	Item	Recommendation	Reported in
Title and abstract	1 (a)	Study design indicated in the title or abstract	Title; Abstract: described as a multisite questionnaire-based cross-sectional study
	1 (b)	Informative and balanced abstract summary	Abstract: structured as Background/Objectives, Methods, Results, Conclusions
Introduction	2	Background and rationale	Section 1
	3	Objectives and prespecified hypothesis	Section 1, final paragraph: descriptive and exploratory objectives; no prespecified statistical hypothesis
Methods	4	Key elements of study design	Section 2.1
	5	Setting, locations, and relevant dates of recruitment and data collection	Section 2.1: dental colleges and affiliated facilities in Riyadh, Al Majma'ah and Jazan; July 2022 to June 2023
	6 (a)	Eligibility criteria, sources and methods of selection of participants	Section 2.2
	7	Outcomes, exposures, predictors and effect modifiers defined	Sections 2.3 and 2.4
	8	Sources of data and methods of assessment	Sections 2.3 and 2.4; full instrument in Appendix A and item provenance in Table S1
	9	Efforts to address potential sources of bias	Section 2.2 (duplicate screening); Section 4.6 (convenience sampling, site confounding, unmodelled clustering, self-report and social desirability bias)
	10	How the study size was arrived at	Section 2.2: no a priori sample-size calculation; convenience sample from the participating institutional networks, whose denominators were not available
	11	Handling of quantitative variables and groupings	Sections 2.3 and 2.4: primary seven-item knowledge score (0–7), with the full 12-item score as a sensitivity analysis; age in three categories; clinical-experience items analysed individually, with 'do not remember' retained as a separate category
	12 (a)	Statistical methods, including control of confounding	Section 2.5: Mann–Whitney U and Kruskal–Wallis tests; multivariable linear regression with HC3 robust standard errors, with all four candidate predictors retained
	12 (b)	Methods for examining subgroups and interactions	Section 2.5: Pearson chi-square tests for item-level subgroup comparisons, with Benjamini–Hochberg false-discovery-rate correction within each table family (Tables 2–5)
	12 (c)	How missing data were addressed	Sections 2.2 and 2.5: isolated blank cells treated as missing values; item-level analyses used available responses and composite scores used complete cases, without imputation (Table S6)
	12 (d)	Sampling strategy, if applicable	Section 2.2: non-probability convenience sampling
	12 (e)	Sensitivity analyses	Sections 2.5, 3.4 and 3.5; Tables S2–S5: full 12-item score, six-item score excluding B7, Poisson model, and qualification analysis restricted to respondents with confirmed

			status; all defined post hoc
Results	13 (a)–(c)	Numbers of participants at each stage; reasons for non-participation; flow diagram	Section 3.1: 923 records analysed, of which 920 were complete across all analysed fields. A participant flow diagram could not be constructed because the survey link was distributed openly and the number invited is unknown (Section 2.2)
	14 (a)	Characteristics of participants and information on exposures	Section 3.1 and Table 1
	14 (b)	Number of participants with missing data for each variable	Sections 3.2 and 3.3; Table S6: one missing value each for B7, B8, B9, C1, C2 and C4; two for B11 and C5; three for B12
	15	Outcome events or summary measures	Sections 3.2–3.4; Tables 2–6
	16 (a)	Unadjusted and adjusted estimates with confidence intervals	Section 3.5 and Table 7
	16 (b)	Category boundaries for continuous variables	Sections 2.3 and 2.4; Table 6
	16 (c)	Translation of relative risk into absolute risk	Not applicable
	17	Other analyses performed	Sections 3.2 to 3.5; Tables S2–S5: FDR-adjusted item-level comparisons and sensitivity analyses
Discussion	18	Key results with reference to study objectives	Section 4.1
	19	Limitations, including sources of bias and imprecision	Section 4.6
	20	Cautious overall interpretation	Sections 4.1–4.5; Section 5
	21	Generalisability of the results	Section 4.6: convenience sample, not representative of the national dental workforce
Other information	22	Funding	Funding statement: no external funding
