# Supplementary material for: Knowledge and Self-Reported Clinical Experience of Oral Potentially Malignant Disorders Among Dental Trainees and General Dentists in Saudi Arabia: A Cross-Sectional Study
Source: Healthcare (Basel). 2026 Oct 15;14(20):3463. doi: 10.3390/healthcare14203463 (PMC13704271; doi:10.3390/healthcare14203463)
Supplement: Supplementary file 1 [file healthcare-14-03463-s001.zip › File S2 CHERRIES checklist.pdf]

File S2. Completed CHERRIES checklist

Checklist for Reporting Results of Internet E-Surveys

Manuscript ID: healthcare-4583004
Oral Potentially Malignant Disorders: Knowledge and Self-Reported Clinical Experience Among Dental Trainees and General Dentists in Saudi Arabia

Item descriptors are summarised. Locations refer to sections, tables and appendices of the revised manuscript.

**Category**	**Checklist item**	**How addressed**
Design	Describe survey design	Multicentre cross-sectional web-based survey; non-probability convenience sample (Sections 2.1 and 2.2)
IRB approval and informed consent process	IRB approval	Approved by the Research Ethics Committee of Riyadh Elm University (protocol SPR/2022/102/761/721, approved 17 July 2022; Section 2.6)
	Informed consent	Purpose, voluntary nature and confidentiality explained on the introductory page; electronic informed consent obtained before access to the items (Section 2.6)
	Data protection	No direct identifiers retained in the analytical dataset; sign-in information available during data cleaning was used only to screen for duplicate submissions (Sections 2.2 and 2.6)
Development and pre-testing	Development and testing	Items adapted from published instruments; content validity reviewed by three faculty members; pilot tested on 30 participants excluded from the final sample (Section 2.3)
Recruitment process	Open survey versus closed survey	Open survey: the link was circulated through institutional mailing lists and messaging groups (Section 2.2)
	Contact mode	Institutional electronic mail lists and messaging groups (Section 2.2)
	Advertising the survey	Distributed through participating institutional networks throughout the recruitment period (Section 2.2)
Survey administration	Web or e-mail	Web-based, administered in Google Forms (Section 2.2)
	Context	Dental colleges and affiliated clinical facilities in Riyadh, Al Majma'ah and Jazan, Saudi Arabia (Section 2.1)
	Mandatory or voluntary	Participation was voluntary and unremunerated. The form was configured to require responses, but the final analytical file contains isolated blank cells whose origin could not be reconstructed; these are handled as missing values (Section 2.2; Table S6).
	Incentives	None offered (Section 2.2)
	Time and date	July 2022 to June 2023 (Section 2.1)
	Randomisation of items or questionnaires	Not used; items were presented in a fixed order
	Adaptive questioning	Not used; all participants saw all items
	Number of items	17 items plus 4 participant characteristics (Section 2.3; Appendix A)
	Number of screens (pages)	Introductory consent page followed by the questionnaire items
	Completeness check	The form was configured with required items; nevertheless, the final analytical file contained isolated blanks (Table S6). Of 923 analysed records, 920 (99.7%) were complete across all analysed fields.
	Review step	Responses could not be revised once submitted (Section 2.2)
Response rates	Unique site visitor	Not recorded; the survey platform did not log unique visitors

	View rate	Not calculable: the number of individuals who viewed the link could not be enumerated (Section 2.2)
	Participation rate	Not calculable for the same reason (Section 2.2)
	Completion rate	A platform-based completion rate cannot be calculated because the number who opened/started but did not submit was not logged. Among the 923 analysed records, 920 (99.7%) were complete across all analysed fields.
Preventing multiple entries	Cookies used	No
	IP check	No
	Log file analysis	No
	Registration	Respondent sign-in was required, but the single-response restriction was not enabled. Duplicate submissions were screened during cleaning; the final file does not retain the screening log, so the number removed cannot be reconstructed.
Analysis	Handling of incomplete questionnaires	No questionnaire was excluded solely for isolated item-level missingness. Item-level analyses used available responses; composite scores used complete cases without imputation (seven-item n=921; full 12-item n=920).
	Questionnaires submitted with an atypical timestamp	Not assessed
	Statistical correction	None applied; no weighting or post-stratification was used
