# Supplementary material for: Knowledge and Self-Reported Clinical Experience of Oral Potentially Malignant Disorders Among Dental Trainees and General Dentists in Saudi Arabia: A Cross-Sectional Study
Source: Healthcare (Basel). 2026 Oct 15;14(20):3463. doi: 10.3390/healthcare14203463 (PMC13704271; doi:10.3390/healthcare14203463)
Supplement: Supplementary file 1 [file healthcare-14-03463-s001.zip › File S3 CROSS checklist.pdf]

File S3. Completed CROSS checklist

Consensus-Based Checklist for Reporting of Survey Studies

Manuscript ID: healthcare-4583004
Oral Potentially Malignant Disorders: Knowledge and Self-Reported Clinical Experience Among Dental Trainees and General Dentists in Saudi Arabia

Item descriptors are summarised and grouped by reporting domain. Locations refer to sections, tables and appendices of the revised manuscript.

Section	Checklist item	How addressed
Title and abstract	Title identifies the study as a survey	The title describes the study population and outcomes; the Abstract explicitly identifies the design as a multisite questionnaire-based cross-sectional study
	Abstract provides a structured summary	Abstract
Introduction	Background and rationale	Section 1
	Purpose, aims and research questions	Section 1, final paragraph
Methods	Study design	Section 2.1
	Data collection methods and setting	Section 2.1 and 2.2: web-based questionnaire administered in Google Forms
	Sample characteristics and sampling technique	Section 2.2: non-probability convenience sampling; eligibility criteria stated
	Sample size and justification	Section 2.2: no a priori calculation; 923 responses analysed
	Survey instrument: description and administration	Section 2.3; full instrument in Appendix A; item provenance in Table S1
	Instrument validity and reliability	Section 2.3: qualitative content validity review by three faculty members; no content validity index computed. Sections 2.4 and 3.4: internal consistency quantified with KR-20 (0.68 for the seven-item score; 0.74 for the full 12-item score)
	Pretesting or piloting	Section 2.3: pilot tested on 30 participants excluded from the final sample
	Recruitment process, period and mode	Section 2.2: institutional mailing lists and messaging groups, July 2022 to June 2023
	Ethical considerations, approval and consent	Section 2.6: Riyadh Elm University REC, protocol SPR/2022/102/761/721, approved 17 July 2022; electronic informed consent obtained
	Data entry, cleaning and management	Section 2.2: duplicate submissions were screened during cleaning; the final file does not retain the screening log, so the number removed cannot be reconstructed. The final analytical file contains isolated blank cells whose origin could not be reconstructed; these are treated as missing (Table S6).
	Statistical methods, including handling of missing data	Section 2.5: Pearson chi-square tests with BH-FDR correction for item-level comparisons; Mann–Whitney/Kruskal–Wallis for score comparisons; HC3 multivariable linear regression; item-level available-case analysis; composite-score complete-case analysis without imputation; sensitivity analyses include 12-item, six-item, Poisson and confirmed-status models.
	Weighting or adjustment procedures	None applied
Results	Response rate and how it was calculated	Section 2.2: not calculable because the number of individuals reached could not be enumerated
	Characteristics of respondents	Section 3.1 and Table 1

	Main findings reported with appropriate precision	Sections 3.2–3.5; Tables 2–7 and Supplementary Tables S2–S6. Denominators reflect available responses; primary knowledge score is based on 921 complete cases.
	Non-response analysis	Not possible; the number of individuals reached could not be enumerated and the characteristics of non-responders are unknown (Sections 2.2 and 4.6)
Discussion	Interpretation of results in context	Sections 4.1–4.5
	Limitations	Section 4.6: includes convenience sampling, unmeasured clustering/site confounding, self-report, unverified “others” qualification category, isolated missing values, post-hoc score definition and measurement limitations.
	Generalisability	Section 4.6: convenience sample; findings not generalisable to the national dental workforce
Other information	Funding and role of funders	Funding statement: no external funding
	Conflicts of interest	Conflicts of Interest statement: none declared
	Data availability	Data Availability Statement
