# Supplementary material for: Knowledge and Self-Reported Clinical Experience of Oral Potentially Malignant Disorders Among Dental Trainees and General Dentists in Saudi Arabia: A Cross-Sectional Study
Source: Healthcare (Basel). 2026 Oct 15;14(20):3463. doi: 10.3390/healthcare14203463 (PMC13704271; doi:10.3390/healthcare14203463)
Supplement: Supplementary file 1 [file healthcare-14-03463-s001.zip › Table S1 questionnaire item provenance.pdf]

Table S1. Provenance and basis of each questionnaire item

Supplementary material

Manuscript ID: healthcare-4583004
Oral Potentially Malignant Disorders: Knowledge and Self-Reported Clinical Experience Among Dental Trainees and General Dentists in Saudi Arabia

The knowledge items (B1–B12) and the clinical experience and perception items (C1–C5) were adapted from previously published questionnaires addressing oral cancer and OPMD awareness among dental populations [16,17,24,25]; the participant characteristics (A1–A4) are standard descriptors. Adaptation consisted of standardising item wording for the present population, presenting every item in a single-best-answer format, and, for most items, providing an explicit "I do not know", "do not remember" or "not sure" option so that uncertainty was not forced into a substantive response (items B3 and B12 had no such option; Appendix A). Items B2, B3, B6, B10 and B12 were excluded from the primary seven-item knowledge score (Section 2.4) but are retained here and in Appendix A for transparency. Reference numbers correspond to the reference list of the main manuscript.

Item	Construct assessed	Source instrument	Designated response	Basis for the designated response
A1–A4	Participant characteristics: sex, age group, recorded level, institutional affiliation	Standard descriptors; not adapted from a source instrument	Not applicable	Standard sociodemographic descriptors. Response options as administered: sex (male, female); age group (20–25, 26–30, >30 years); level (last-year dental student, dental intern, dental general practitioner, others); place of study or work (government, private). For the primary qualification analyses, all non-GDP levels were grouped as trainees (Section 2.3).
B1	Whether OPMDs invariably progress to malignancy	Adapted from [16,17,24,25]	No	Malignant transformation is a risk, not an inevitability; pooled transformation estimates range from about 1.4% for oral lichen planus to 49.5% for proliferative verrucous leukoplakia [8].
B2	Whether oral epithelial dysplasia resolves spontaneously	Adapted from [16,17,24,25]	No	Dysplasia may persist, progress or regress, and regression does not abolish subsequent risk; the dichotomous framing is acknowledged as a limitation in Section 4.6 [6,7]. Excluded from the primary score (Section 2.4).
B3	Interval after which an unexplained oral lesion requires definitive assessment	Adapted from [16,17,24,25]	More than 2 weeks	The two-week convention for an unexplained persistent oral lesion is widely applied in referral guidance and underpins early-detection pathways [4,5]. Excluded from the primary score (Section 2.4).
B4	Most frequently encountered OPMD	Adapted from [16,17,24,25]	Leukoplakia	Leukoplakia is the most prevalent OPMD in most reported series [6,7].
B5	Meaning of the term leukoplakia	Adapted from [16,17,24,25]	White lesion	Tests recognition of the descriptive basis of the term; the item does not test the full diagnostic-of-exclusion definition, which is noted in Section 4.2 [6].
B6	Commonest site of oral erythroplakia	Adapted from [16,17,24,25]	Tongue	Floor of mouth, lateral tongue, soft palate and retromolar region are all described as characteristic sites and reported distributions vary between series; the single-best-answer format is acknowledged as a limitation in Section 4.6 [28–30]. Excluded from the primary score (Section 2.4).
B7	Lesion most likely to show dysplasia	Adapted from	Erythroplakia	Erythroplakia carries the highest reported malignant transformation and

	or carcinoma	[16,17,24,25]		dysplasia yield among the lesions offered as response options [8,30]. Retained in the primary score; the wording does not distinguish per-lesion risk from population-attributable burden (Sections 2.4 and 4.2).
B8	Subtype of oral lichen planus with greatest malignant potential	Adapted from [16,17,24,25]	Erosive	Erosive and atrophic forms carry elevated risk relative to reticular disease on systematic review [32].
B9	Recognition of homogeneous leukoplakia from its clinical description	Adapted from [16,17,24,25]	Homogeneous leukoplakia	Corresponds to the accepted clinical description of a uniformly flat, thin, white lesion with shallow surface cracks [6,7].
B10	Principal risk factor for malignant transformation	Adapted from [16,17,24,25]	Site of the lesion	Site is a recognised predictor, although non-homogeneous phenotype, lesion size, tobacco exposure and epithelial dysplasia are also established predictors; the single-best-answer format is acknowledged as a limitation in Section 4.6 [8]. Excluded from the primary score (Section 2.4).
B11	Recognition of oral submucous fibrosis from its presenting symptoms	Adapted from [16,17,24,25]	Oral submucous fibrosis	Burning sensation and intolerance of spicy food are characteristic early symptoms of oral submucous fibrosis [6,7].
B12	Investigation required for definitive diagnosis	Adapted from [16,17,24,25]	Histopathological examination	Histopathology remains the reference standard for definitive diagnosis of a suspicious oral lesion; the item is framed broadly and this is acknowledged in Section 4.6 [14]. Excluded from the primary score (Section 2.4).
C1–C5	Self-reported clinical experience and perceptions: lesion detection, referral, biopsy, perceived difficulty, appropriate clinician	Adapted from [16,17,24,25]	Not applicable (no designated correct response)	Reported item by item as frequencies; no composite score was calculated because the items assess distinct dimensions (Section 2.4).

The four source questionnaires [16,17,24,25] were used collectively in assembling the instrument; individual items were not drawn verbatim from a single source, and the adaptation described above was applied across items.
