# Supplementary material for: Knowledge and Self-Reported Clinical Experience of Oral Potentially Malignant Disorders Among Dental Trainees and General Dentists in Saudi Arabia: A Cross-Sectional Study
Source: Healthcare (Basel). 2026 Oct 15;14(20):3463. doi: 10.3390/healthcare14203463 (PMC13704271; doi:10.3390/healthcare14203463)
Supplement: Supplementary file 1 [file healthcare-14-03463-s001.zip › Table S2 to S6 SENSITIVITY ANALYSES HIGHLIGHTED.pdf]

Supplementary Tables S2 to S6: Sensitivity Analyses and Missing-Data Summary

Manuscript ID: healthcare-4583004. All analyses below were performed on the final analytical dataset (n = 923). Item-level analyses use available responses; composite scores use complete cases without imputation.

Reference categories for all regression models: female sex, age 20–25 years, trainee status and government institutional affiliation. Standard errors and 95% confidence intervals are based on HC3 heteroskedasticity-robust standard errors.

Table S2. Side-by-side seven-item and full 12-item multivariable models on the same respondents (n = 920)

Covariate	7-item B	7-item HC3 SE	7-item 95% CI	7-item p	12-item B	12-item HC3 SE	12-item 95% CI	12-item p
Male sex	0.95	0.15	0.65 to 1.24	<0.001	2.07	0.21	1.65 to 2.48	<0.001
Age 26–30 years	-0.72	0.15	-1.01 to -0.43	<0.001	-1.33	0.22	-1.75 to -0.91	<0.001
Age >30 years	-1.77	0.28	-2.33 to -1.22	<0.001	-2.50	0.40	-3.29 to -1.72	<0.001
GDP status	1.50	0.15	1.21 to 1.79	<0.001	2.26	0.22	1.83 to 2.69	<0.001
Private affiliation	-1.12	0.19	-1.49 to -0.75	<0.001	-1.84	0.29	-2.40 to -1.28	<0.001

Model fit: seven-item $R^2 = 0.537$; 12-item $R^2 = 0.629$. All covariates retained the same direction and statistical significance. Coefficient magnitudes are not directly comparable because score ranges differ (0–7 vs 0–12).

Table S3. Six-item sensitivity analysis excluding B7 (n = 921)

Mean = 3.57 ± 1.52 of 6; KR-20 = 0.60; $R^2 = 0.582$.

Covariate	B	HC3 SE	95% CI lower	95% CI upper	p
Male sex	0.73	0.13	0.47	0.99	<0.001
Age 26–30 years	-0.51	0.13	-0.77	-0.26	<0.001
Age >30 years	-1.59	0.25	-2.09	-1.10	<0.001
GDP status	1.34	0.13	1.09	1.59	<0.001
Private affiliation	-1.04	0.16	-1.36	-0.71	<0.001

Table S4. Qualification sensitivity restricted to explicitly identified status

The analysis included 673 respondents: 267 last-year students, 205 interns and 201 GDPs. Identified trainees (n=472) scored 4.60 ± 1.25; GDPs scored 4.82 ± 1.30 (Mann–Whitney p=0.013). Students scored 3.96 ± 0.95; interns 5.42 ± 1.11 (p<0.001). In the adjusted confirmed-status model, the GDP coefficient was +0.64 (95% CI 0.22 to 1.06; p=0.003; R^2=0.070). In this model, male sex, age over 30 years and private affiliation were not statistically significant.

Covariate	B	HC3 SE	95% CI lower	95% CI upper	p
Male sex	0.17	0.20	-0.23	0.58	0.394
Age 26–30 years	-0.89	0.23	-1.35	-0.44	<0.001
Age >30 years	-0.84	0.71	-2.24	0.55	0.237
GDP status	0.64	0.21	0.22	1.06	0.003
Private affiliation	-0.47	0.29	-1.03	0.09	0.102

Table S5. Poisson sensitivity model for the seven-item score (n = 921)

Covariate	Expected-score ratio	95% CI lower	95% CI upper	p
Male sex	1.34	1.23	1.47	<0.001
Age 26–30 years	0.86	0.79	0.93	<0.001
Age >30 years	0.43	0.30	0.61	<0.001
GDP status	1.53	1.40	1.67	<0.001
Private affiliation	0.66	0.57	0.77	<0.001

Table S6. Item-level missing values in the final analytical dataset

Item	Missing values
B7. Lesion most likely to show dysplasia or carcinoma	1
B8. Lichen planus subtype most likely to be malignant	1
B9. Clinical description of homogeneous leukoplakia	1
B11. Presenting symptoms of oral submucous fibrosis	2
B12. Investigation required for definitive diagnosis	3
C1. Has detected a lesion suspicious for oral cancer	1
C2. Has referred a patient to a specialist	1
C4. Experiences difficulty in identifying OPMDs	1
C5. Considers an oral medicine or oral pathology specialist the appropriate clinician to detect OPMDs	2

Overall, 920/923 records (99.7%) were complete across all analysed fields. The primary seven-item score used 921 complete cases; the full 12-item sensitivity score used 920; the six-item sensitivity score used 921. No missing-value imputation was used. Item codes follow Appendix A of the manuscript.
